# Molecular Weight Analysis of Blue Shark (*Prionace glauca*) Collagen Hydrolysates by GPC-LS; Effect of High Molecular Weight Hydrolysates on Fibroblast Cultures: mRNA Collagen Type I Expression and Synthesis

**DOI:** 10.3390/ijms23010032

**Published:** 2021-12-21

**Authors:** María Blanco, Noelia Sanz, Ana C. Sánzhez, Begoña Correa, Ricardo I. Pérez-Martín, Carmen G. Sotelo

**Affiliations:** Food Biochemistry Group, Instituto de Investigaciones Marinas, Consejo Superior de Investigaciones Científicas, C/Eduardo Cabello, 6, CP 36208 Vigo, Spain; nsanz@iim.csic.es (N.S.); asanchez@iim.csic.es (A.C.S.); begonacorrea@iim.csic.es (B.C.); ricardo@iim.csic.es (R.I.P.-M.); carmen@iim.csic.es (C.G.S.)

**Keywords:** fish by-products, collagen, fish protein hydrolysates, molecular weight, GPC-LS, fibroblast, mRNA collagen expression, pro-collagen

## Abstract

High molecular weight (Mw) collagen hydrolysates have been demonstrated to produce a higher synthesis of collagen type I mRNA. Mw determination is a key factor maximizing the effect of collagen hydrolysates on collagen type I synthesis by fibroblasts. This work aimed to achieve a high average Mw in Blue Shark Collagen Hydrolysate, studying different hydrolysis parameters by GPC-LS analysis and testing its effect on mRNA Type I collagen expression. Analysis revealed differences in blue shark collagen hydrolysates Mw depending on hydrolysis conditions. Papain leads to obtaining a significantly higher Mw hydrolysate than Alcalase at different times of hydrolysis and at different enzyme/substrate ratios. Besides, the time of the hydrolysis factor is more determinant than the enzyme/substrate ratio factor for obtaining a higher or lower hydrolysate Mw when using Papain as the enzyme. Contrary, Alcalase hydrolysates resulted in similar Mw with no significant differences between different conditions of hydrolysis assayed. Blue shark collagen hydrolysate showing the highest Mw showed neither cytotoxic nor proliferation effect on fibroblast cell culture. Besides, it exhibited an increasing effect on both mRNA expression and pro-collagen I production.

## 1. Introduction

Marine collagen is mainly obtained from the skin of different Teleost and Chondrichthyes species but also from invertebrates (sponges or jellyfish) and more recently, from fish by-products generated by the fishery industry (fins, bones, or scales) [1]. Marine collagen has produced an increasing interest in recent decades as it presents excellent functional and safe (low antigenicity) properties while at the same time meeting several requirements of different types of consumers (Halal, Kosher, Pescatarians, etc.). Marine collagen-derived peptides, produced by controlled hydrolysis of collagen, has also experienced an increasing interest as many recent publications revealed their benefits and excellent bioactivities such as antioxidant, anti-inflammatory, reduction of melanin synthesis, tyrosinase, or matrix metalloproteinase inhibition, with applications in the development of anti-aging and anti-skin wrinkles cosmeceuticals [1].

Enzymatic hydrolysis of proteins produces different profiles of peptides with different properties and bioactivities, from the same raw material, only by varying the hydrolysis conditions (temperature, enzyme, time of hydrolysis, or enzyme/protein ratio) [2]. The resultant hydrolysates comprise a wide range mixture of peptides which might vary from very high to very low molecular weight (Mw) peptides. Mw analysis of collagen hydrolysates is of key importance as previously published results indicated that higher molecular weight collagen peptides produce a higher synthesis of collagen type I mRNA in human dermal fibroblast [3]. SDS-PAGE and HPLC-MS are methodologies extensively employed to determine the Mw profile of protein hydrolysates. However, these techniques do not provide enough Mw resolution (both accuracy and quantitation) for the analysis of hydrolysates that result from a wide range of molecular weights, representing a drawback as has been already pointed out [3]. Besides, those methodologies are time-consuming, and many sample handling steps are required. To provide an in-depth, innovative, and more resolute analysis of collagen hydrolysates Mw, gel permeation chromatography (GPC) using multi-detector was studied. Single concentration detector such as refractive index (IR) or ultraviolet light (UV) used in conventional GPC gives limited information regarding how much material is eluted from the column at a given time, data which is converted into an Mw distribution by reference to a calibration curve, assuming that a certain size in solution corresponds to a certain Mw. However, a wrong Mw could be determined if the chemistry of the peptide standards used in the calibration and the samples are different, since different molecules (peptides) with different chemistry coil up differently in solution and this might affect their elution time. Multidetector GPC employing a concentration detector (refraction index; IR) and a light scattering detector (LS) addresses the limitation of conventional GPC. Besides, GPC-LS enables the determination of concentration analysis and absolute determination of Mw of peptides and proteins from 10 kDa, up to big proteins such as thyroglobulin with a molecular mass of 670 kDa [4]. For the reasons explained above, GPC-LS is a powerful method for analyzing and determining the Mw of polymers [5,6], however and to our knowledge, its use for determining the Mw of collagen hydrolysates is still null. The main objective of this work was to obtain a high collagen hydrolysate Mw from blue shark skin by-products: Blue shark collagen hydrolysate (BSCH), studying different hydrolysis parameters by means of gel permeation chromatography coupled to a light scattering detector (GPC-LS). After, the bioactivity of the selected collagen hydrolysate was evaluated by measuring mRNA Type I collagen expression and collagen Type I synthesis in human dermal fibroblasts cultures.

## 2. Results and Discussion

### 2.1. Collagen Extraction

The three-step collagen extraction procedure was repeated several times until achieving the required amount of Blue Shark Acid Soluble Collagen (BSASC) needed to perform the collagen hydrolysis experiments. The proximate composition of lyophilized BSASC is shown in Table 1. The yield in each collagen extraction procedure was 4.72% (g collagen/ 100 g dried skin), which is a similar value to that obtained previously for Blue shark PSC [7].

#### 2.1.1. SDS-PAGE

**Figure 1:**
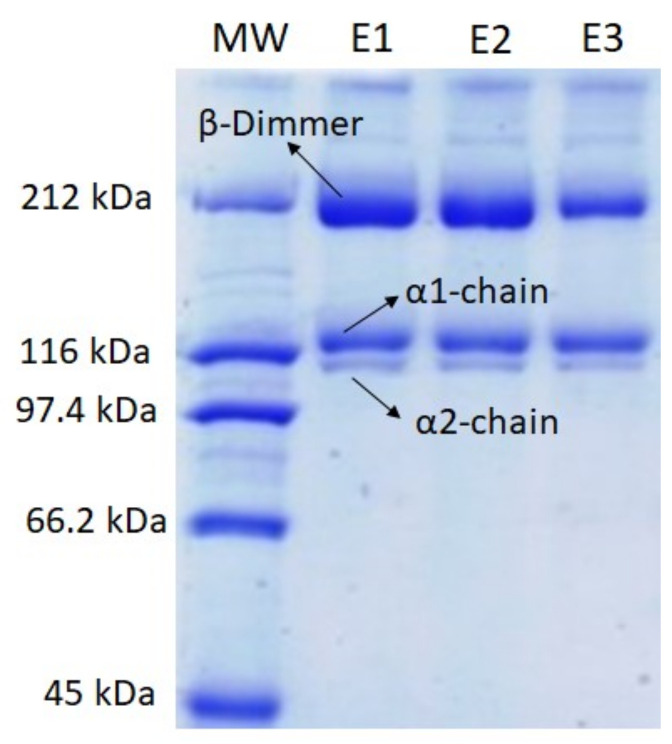
SDS-PAGE profile of Blue shark collagen obtained in three sequential extraction steps (E1, E2, E3).

To check if the subunit composition (α and β components) of BSASC varied in the three extraction steps (E1, E2, and E3), the protein pattern of collagen obtained in each extraction step was analyzed (Figure 1). The results showed no differences between the subunit composition of the collagen obtained in the three sequential steps. The unique minor difference was a faint β band in the last extraction step (E3) compared with those obtained in E1 and E2 steps, which might reflect a lower number of crosslinked components in the collagen obtained in this last step. 

### 2.2. Effect of Hydrolysis Conditions on BSCH Mw

#### 2.2.1. GPC-LS Analysis

The average Mw determined by GPC-LS was analyzed in the 24 hydrolysates in order to select those conditions of hydrolysis leading to obtaining the highest Mw. Table 2 shows the Mw of Blue Shark Collagen Hydrolysates (BSCH) obtained after GPC-LS analysis which were prepared under different conditions. Results showed that the type of enzyme selected is the main factor determining the Mw of the resultant hydrolysate. Thus, Mw observed for Papain hydrolysates (experiments A, C, F, and G) was significantly higher than that obtained for Alcalase ones (experiments D, H, B, and E) (4.78 ± 2.45 for Alcalase hydrolysates and 19.02 ± 8.27 for Papain hydrolysates; Kruskal–Wallis test *p* = 0.005). The Kruskal–Wallis test did not reveal significant differences between the Mw obtained using 1/20 or 1/5 E/S ratio, nor between the Mw obtained after 0.5 h or 6 h time of hydrolysis. However, the analysis of the effect of time of hydrolysis within Papain hydrolysates showed that the hydrolysis for 30 min (experiments C and F) led to obtaining a significantly higher Mw using both an enzyme substrate ratio (E/S) of 1/20 or 1/5, compared to 6 h of hydrolysis (experiments A and G) (27.57 ± 2.44 for 0.5 h hydrolysis time and 10.46 ± 3.38 for 6 h hydrolysis time; Kruskal–Wallis test *p* < 0.05). Therefore, a lower time of hydrolysis in combination with either a low or high E/S ratio led to obtaining higher Mw hydrolysates for Papain. The analysis of the effect of E/S ratio in the Mw obtained within Papain hydrolysates showed no significant differences between 1/5 and 1/20 E/S ratios at both times of hydrolysis. Therefore, it could be concluded that the time of hydrolysis factor is more determinant than the E/S factor for obtaining a higher Mw hydrolysate when using Papain as the enzyme.

Regarding the analysis of Alcalase hydrolysates, Kruskal–Wallis test revealed no significant differences between the Mw obtained using 1/20 or 1/5 E/S ratios nor using 0.5 h or 6 h of time of hydrolysis.

**Figure 2:**
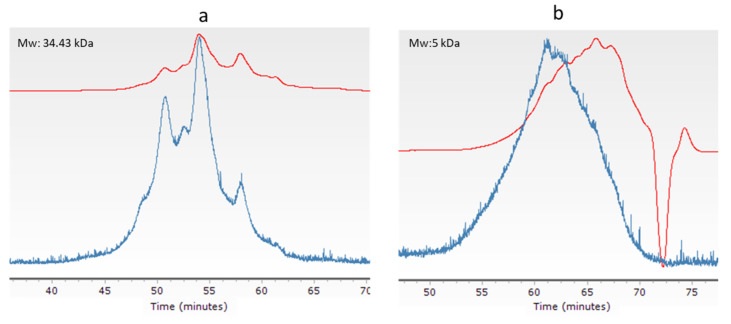
GPC profile of hydrolysates selected for further cell viability and mRNA type I collagen expression analysis showing their respective molecular weight: BSCH-C (**a**) and CHC (**b**). Blue line: 90° light-scattering signal (LS); red line: refractive index signal (RI).

Figure 2 shows the GPC-LS profile of the highest Mw BSCH, which were selected for further cell viability studies (BSCH-C) besides a commercial hydrolyzed collagen (CHC). The CHC was also analyzed in triplicate by GPC-LS for comparative purposes showing an Mw of 5.3 ± 0.17 kDa which was similar to Alcalase hydrolysates.

GPC-LS resulted in being a useful technique for determining differences in Mw of BSCH due to different hydrolysis conditions and to compare it with a commercial hydrolyzed collagen. 

#### 2.2.2. HPLC-SEC Analysis

**Figure 3:**
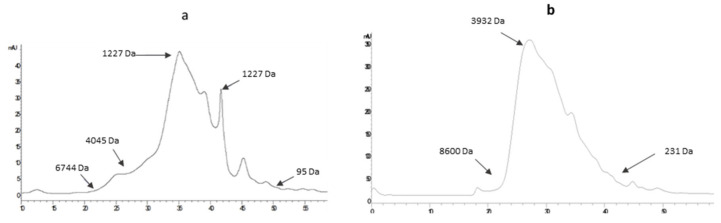
Molecular weight profile of BSCH-C (**a**) and CHC (**b**) obtained using SEC technique.

The molecular weight was also determined by HPLC-SEC in the 24 hydrolysates to cover and study the whole range of Mw of each hydrolysate as the GPC-LS analysis developed is only reliable for Mw over 10 kDa. HPLC-SEC allows a high-resolution molecular weight detection of peptides between 0.1–7 kDa, therefore, covering the lack of low molecular weight resolution of GPC-LS analysis. Figure 3 shows the Mw profile of BSCH-C and CHC, which were further analyzed in terms of cell viability and mRNA collagen expression assays. Table 3 shows the molecular weight distribution obtained after HPLC-SEC analysis, where it can be observed that different conditions of hydrolysis lead to obtaining different Mw distribution profiles. The higher percentage of greater Mw peptides (17–3 kDa) was observed in Papain hydrolysates (experiments **A**, **C**, **F**, and **G** with values of 17, 9, 18, and 30%, respectively). While Alcalase hydrolysates, experiments **B**, **D**, **E**, and **H**, showed lower values of those high Mw peptides (2, 4, 3, and 2%, respectively). These results agree with those found using GPC-LS analysis. Although HPLC-SEC revealed that Papain and Alcalase hydrolysates presented a similar percentage of low Mw peptides, it is noteworthy the highest percentage of low Mw peptides was found in BSCH-C (9%).

### 2.3. Amino Acid Characterization of BSCH-C and CHC

The BSCH-C and CHC were analyzed in terms of amino acid content for comparison studies and also to determine if amino acid differences between both hydrolysates might influence differences in collagen production by human dermal fibroblast in vitro (Table 4). Results showed that BSCH-C was rich in glycine (306 residues/1000 residues), followed by alanine (118 residues/1000 residues), proline (112 residues/1000 residues), glutamic acid (76 residues/1000 residues), and amino acids as it was expected from a collagen hydrolysate [7]. The main difference between BSCH-C and CHC was the higher % hydroxyproline content in the latter and the consequent higher amount of all the other amino acids in the BSCH with the exemption of hydroxylysine and proline which were lower. 

### 2.4. Effect of HMW-BSCH and CHC on Cell Viability

**Figure 4:**
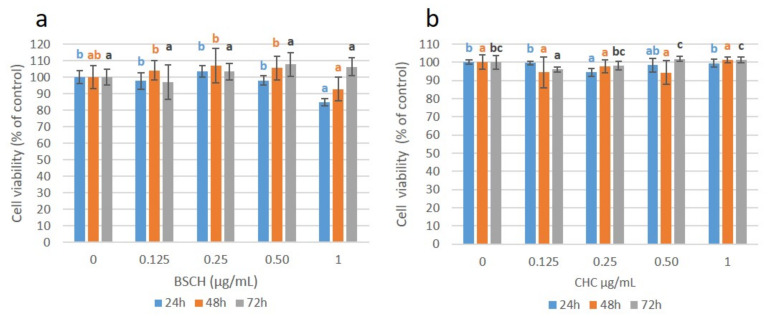
Cell viability values (%) for the fibroblast cell cultures treated with BSCH (**a**) and CHC (**b**) hydrolysates and normalized to the control group. Values are shown as means with standard deviation. Different letters indicate a significant difference between concentration groups (*p* < 0.05).

The effect of BSCH and CHC on cell viability was tested with the aim of comparative analysis. As can be seen in Figure 4, cell viability was not substantially decreased by the addition of both collagen hydrolysates. For all the hydrolysates concentrations tested (0.125 to 1 mg/mL), the percentage of viability remains above the threshold of 70%, which is considered the limit for cell cytotoxicity [8].

These results suggest the efficacy of using Papain with an enzyme/substrate ratio of 1/20 for 30 min to obtain a non-cytotoxic BSCH with cell viability values similar to those of commercial collagen hydrolysate and comparable to results obtained by other authors [9,10,11,12].

### 2.5. Effect of High Mw BSCH and CHC on mRNA Collagen Synthesis of Fibroblast Culture

**Figure 5:**
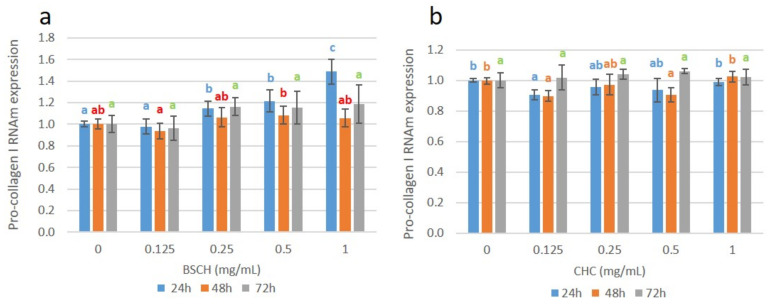
Pro-collagen I RNAm expression for the fibroblast cell cultures treated with BSCH-C (**a**) and CHC (**b**) hydrolysates and normalized to the control group. Values are shown as means with standard deviation. Different letters indicate significant differences between concentration groups (*p* < 0.05).

Collagen expression was investigated at the gene level by determining the mRNA expression of the Pro-collagen I gene (COL_I) in a fibroblast cell culture incubated with BSCH-C and CHC hydrolysates (Figure 5a,b).

Cell culture treated with BSCH-C significantly enhanced COL_I RNA expression at 24 h for the 0.25, 0.5, and 1 mg/mL concentrations. An increased tendency was observed, reaching a value of relative gene expression of 1.5 for the concentration of 1mg/mL. Similar results have been obtained by other authors [13,14]. In contrast, this dose-response effect was not observed for the cell cultures treated with BSCH-C during 48 and 72 h [15] previously observed similar behavior in fibroblast cell cultures treated with collagen hydrolysates from bovine skin. Likewise, no dose-response effect was observed for the fibroblast treated with the CHC hydrolysate at any time. 

### 2.6. Effect of HMW-BSCH and CHC on Pro-Collagen I Synthesis (ELISA) of Fibroblast Culture

**Figure 6:**
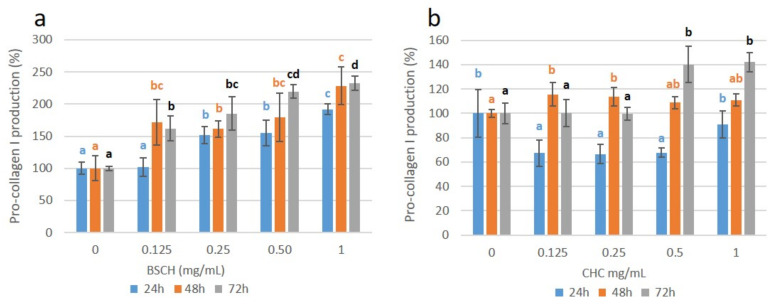
Pro-collagen I production for the fibroblast cell cultures treated with BSCH-C (**a**) and CHC (**b**) hydrolysates and normalized to the control group. Values are shown as means with standard deviation. Different letters indicate significant differences between concentration groups (*p* < 0.05).

**Figure 7:**
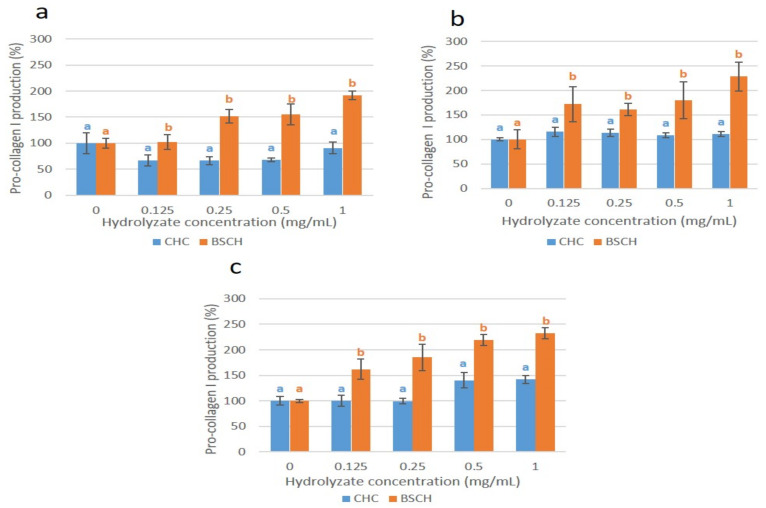
Effect of BSCH-C (orange) and CHC (blue) hydrolysates on collagen production enhancement of fibroblast cell cultures at 24 (**a**), 48 (**b**) and 72 (**c**) h. Values are shown as means with standard deviation. Different letters indicate a significant difference between concentration groups (*p* < 0.05).

A gradual overexpression of pro-collagen I was observed in normal human dermal fibroblasts treated with BSCH-C at 24 h of incubation as the concentration of the treatment increased (Figure 6a), coinciding with the overexpression of the COL_I mRNA (strong correlation; r = 0.787 **; (**: correlation is significant at *p* < 0.01 level). Such overexpression was maintained at 48 h and increased significantly again at 72 h of incubation, reaching 2.3 times for the fibroblasts treated with 1 mg/mL of hydrolysate compared with the untreated fibroblasts. This effect was not appreciated in the cell culture treated with the CHC, since at 24 h, an under-expression of pro-collagen I production was observed. At 48 h of incubation, a slight overexpression for all the concentrations tested, without significant differences between them (Figure 6b) was appreciated. After 72 h of incubation, the cell culture treated with the CHC at 0.5 and 1 mg/mL concentrations showed a pro-collagen I overexpression, reaching 1.4 times higher than that of untreated cells. As can be seen in Figure 7, the pro-collagen I overexpression produced by the cells treated with the BSCH-C hydrolysate was higher than that achieved by the cells treated with the CHC for all the concentrations tested as well as for all the incubation periods (24, 48, and 72 h), reaching more than the double for 3 of the 4 concentrations tested (0.25, 0.5, and 1 mg/mL) at 24 h and for the concentration of 1 mg/mL at 48 h.

Such increased productions were not due to an increase in cell number, as non-cell proliferation was observed, but rather to activation of cell metabolism related to collagen biosynthesis. Therefore, the increased collagen production displayed by BSCH-C, compared to CHC, might be linked to the effect of its particular amino acid profile (higher percentage of all amino acid except hydroxyproline content) and/or its particular molecular weight profile (higher Mw determined by GPC-LS and the presence of a higher percentage of lower Mw peptides determined by HPLC-SEC), on a cellular mechanism, e.g., a biosynthesis pathway, chaperone synthesis pathway (maintenance of conformation) or inhibition of MMP (collagenolytic matrix metalloproteinases) synthesis [16,17,18]. These results would suggest that the “dynamic reciprocity” model, proposed by Bisell et al. 1982 [19], by which the interaction between ECM molecules and cell receptors give rise to a cascade of intracellular signals that revert to the ECM [20].

## 3. Materials and Methods

### 3.1. Biological Samples and Compositional Analysis

Blue shark (*Prionace glauca*) skins were kindly provided by Protea S.L. (Marín, Pontevedra) and transported to the laboratory at 4 °C. Skins were initially mechanically cut into small pieces (5 × 5 cm^2^), and then each of those pieces were manually cut into smaller pieces (0.5 × 0.5 cm^2^), mixed thoroughly, separated in sealed plastic bags, each containing 300 g of skin, and stored at −20 °C until used for the experiment.

The chemical composition of skins was determined in triplicate by analyzing crude protein, ash, moisture, and fat content. Total nitrogen was determined with the Kjeldahl method [21] in a DigiPREP HT digestor (SCP Science, Baie-d′Urfe, QC, Canada), DigiPREP 500 fully automatic steam distillation (SCP Science, Baie-d´Urfe, QC, Canada), and a TitroLine easy titration unit (Schoot, Mainz, Germany), and crude protein content was calculated as total nitrogen multiplied by 6.25. Fat content was determined by the Bligh and Dyer method [22]. Moisture was determined after heating the sample at 105 °C for 24 h, and ash content was determined after heating the sample at 550 °C for 24 h.

### 3.2. Extraction of Acid Soluble Collagen

An acid extraction collagen procedure was carried out following the methodology of Blanco et al. 2020 [6] to obtain the blue shark acid-soluble collagen (BSASC). Briefly, 300 g of skins were mixed with 10 volumes of 0.1 M NaOH and stirred in a cold room (4 °C) for 24 h. The liquid was discarded, and the NaOH-treated skins were washed with distilled water until neutral pH was achieved. Washed residue was stirred 24 h with 10 volumes of 0.5 M acetic acid, then the extract was centrifuged (3000× *g*, 15 min) and the supernatant was diafiltrated using spiral polyethersulfone membranes of 0.56 m^2^ (Prep/Scale-TFF, Millipore Corporation, Bedford, MA, USA) with 30 kDa molecular weight cut-offs (MWCO). Diafiltrated solutions were then freeze-dried, obtaining the acid-soluble collagen (BSASC-1). The treated skins, which were the residue obtained after the previous centrifugation step, were mixed with 10 volumes of 0.5 M acetic acid for 24 h and centrifuged (3000× *g*, 15 min), obtaining the BSASC-2 (after a diafiltration and freeze-drying step as previously described). The treated skins, which were the residue obtained after the previous centrifugation step were mixed with 10 volumes of 0.5 M acetic acid for 24 h and centrifuged (3000× *g*, 15 min) obtaining the BSASC-3 (after a diafiltration and freeze-drying step as previously described). Total extracted collagen (BSASC) was obtained by mixing BSASC-1, BSASC-2, and BSASC-3. 

#### 3.2.1. Proximate Composition

The chemical composition of BSASC was evaluated in triplicate, analyzing moisture, crude protein, lipids, and ash content according to the details described in Section 3.1.

#### 3.2.2. SDS-PAGE

To check if the subunit composition (α and β components) of BSASC varied in the three extraction steps, the protein pattern of BSASC-1, BSASC-2, and BSASC-3 was analyzed using sodium dodecyl sulphate poly-acrylamide gel electrophoresis (SDS-PAGE) according to the procedure described in Laemmli [23]. Samples (1 mg/mL) were prepared in sample buffer containing 10.52% glycerol, 21% Sodium Dodecyl Sulphate (SDS) (10%), 0.63% Dithiothreitol (DTT), and 0.5 M Tris-HCl (pH 6.8) and heated for 5 min at 100 °C. An aliquot (8 µL) of this mixture was applied to each well in 7% polyacrylamide separating gels (100 mm × 750 mm × 0.75 mm) and subjected to electrophoresis at 20 mA using a Mini-Protean II cell (Bio-Rad, Hercules, CA, USA). Afterwards electrophoresis gels were stained with 0.04% Coomassie Blue in 25% ethanol (*v*/*v*) and 8% acetic acid (*v*/*v*) for 2 h. Excess stain was removed with several washes of destaining solution (25% ethanol (*v*/*v*), 8% acetic acid (*v*/*v*)). Molecular weights of collagen subunits were estimated using a wide-range molecular weight standard (Amresco).

### 3.3. Enzymatic Hydrolysis of Acid Soluble Collagen

**Figure 8:**
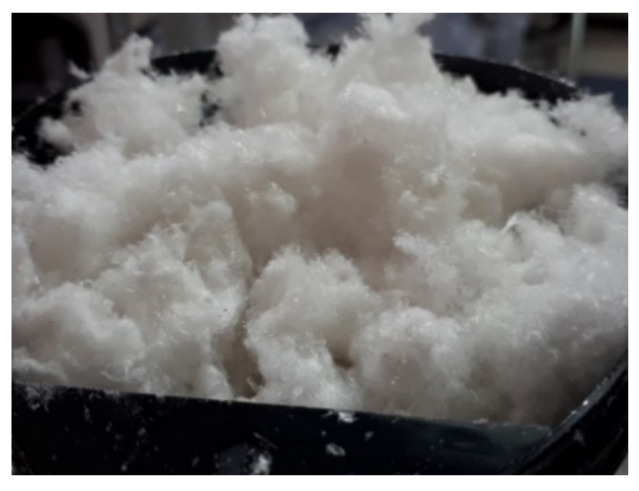
Hydrolyzed collagen obtained in the following conditions: type of enzyme: Papain; E/S: 1/20; time of hydrolysis: 0.5 h).

An experimental design including two different enzymes, Papain (Merck, KGaA, Darmstadt, Germany) and Alcalase 2.4 L (Novozymes, Nordisk, Bagsvaerd, Denmark); different enzyme/substrate ratios (E/S) (1/20 and 1/5); time of hydrolysis (0.5 h and 6 h) was developed with three replicates per condition to study the influence of hydrolysis conditions on the molecular weight of the resultant blue shark collagen hydrolysates (BSCH). The rest of the experimental conditions were maintained constant: solid: liquid ratio (1:100) and 200 rpm of agitation. The randomization of the experiments was done with software www.random.org (accessed on 1 March 2019). Enzymatic hydrolysis of BSASC was carried out in a controlled pH-Stat system with a 100 mL glass reactor. pH levels of each of the 24 points of the experimental design (four different conditions with each enzyme and three replicates for each condition) were adjusted by adding 0.1 M NaOH until the optimal pH of each enzyme (pH 8.0 for Alcalase and pH 7.0 for Papain) was achieved, then the pH was maintained constant during hydrolysis reactions by automatic addition of 0.1 M NaOH. At the end of each hydrolysis, samples were heated at 90 °C for 10 min to inactivate the enzymes, frozen, lyophilized, and stored at −20 °C until analysis (Figure 8). 

### 3.4. Characterization of Hydrolysates

#### 3.4.1. Size-Exclusion Chromatography

The molecular weight distribution of the 24 BSCH and a commercial hydrolyzed collagen (CHC) (obtained from Atlantic cod collagen) was estimated by gel filtration chromatography using an Agilent 1260 Infinity System (Agilent Technologies, Pittsburg, PA, USA) equipped with a Superdex Peptide 10/300 GL column (GE Healthcare, Uppsala, Sweden), with an isocratic elution using 0.1% Trifluoro-acetic acid (TFA) in 30% acetonitrile as the eluent. The samples of freeze-dried hydrolysates were dissolved in the eluent (10 mg/mL), filtered through a 0.45 µm filter, and ultrafiltered using a 10 kDa molecular weight cut-off Amicon Ultra Device. Aliquots of 10 µL were injected, eluted at 0.4 mL/min and monitored at 220 nm. The column was calibrated using the following standard proteins (Sigma, St. Louis, MO, USA): cytochrome C (12,400 Da), aprotinin (6500 Da), angiotensin II (1046 Da), leucine–enkephalin (555 Da), Val–Tyr–Val (379 Da) and Gly–Gln (221 Da).

#### 3.4.2. GPC of Blue Shark Collagen Hydrolysates

The molecular weight of the 24 BSCH and CHC were determined by size exclusion chromatography (SEC) with measurements carried out in an Agilent 1260 HPLC consisting of a G1311B quaternary PUMP, a G1329B injector, a G1316A column oven, a G1362A refractive index (RI) and a dual-angle static light scattering DALS, G7800A detector. For chromatographic separations, four columns were used (PSS, Mainz, Germany): Proteema precolumn (5 µm, 850 mm), Proteema 1000 Å (5 µm, 8300 mm), Proteema 300 Å (5 µm, 8300 mm) and Proteema 100 Å (5 µm, 8300 mm). For sample preparation, lyophilized BSC and BSCH were dissolved in the mobile phase (0.15 M sodium acetate, 0.2 M acetic acid, pH 4.5) (1 mg/mL) and filtered through a PTFE 0.2 µm membrane filter. A total of 100 µL of the filtered solution was injected and chromatographed at an eluent flow rate of 0.5 mL/min using an isocratic elution profile. The column oven was kept at 20 °C, RID at 35 °C and DALS at 30 °C. DALS detector was calibrated with a polyethylene oxide standard (PEO) (PSS, Mainz, Germany) of 106 kDa (Mp) and polydispersity index 1.05. Refractive index increments (dn/dc) were adopted from Meyers and Morgenstern (2003) [24]. Data were analyzed using Agilent GPC/SEC software A.02.01 (Santa Clara, CA, USA).

#### 3.4.3. Statistical Analysis

Statistical analysis was performed with IBM SPSS 26 software (IBM Corporation, Armonk, NY, USA). Prior to analysis, Mw data were checked for normality and homoscedasticity using Kolmogorov–Smirnov and Levene tests, respectively. Kolmogorov–Smirnov showed that molecular weight data and its transformations did not fit with the assumptions of normality. Then, a non-parametric Kruskal–Wallis test was used for data analysis. *p*-values < 0.05 were considered statistically significant.

### 3.5. Amino Acid Characterization of BSCH and CHC

Amino acids were determined in the BSCH, which showed higher molecular weight in the commercial hydrolyzed collagen. For this purpose, acid hydrolysis was performed using 6 N HCl containing 0.1% phenol under an inert atmosphere by heating at 110 °C for 24 h. Then, HCl was removed by vacuum. The hydrolysate was resuspended in 20–50 µL of 0.2 M sodium citrate buffer (pH 2.2), to which a known amount of norleucine was added as an internal standard and applied to an automated amino acid analyzer (Biochrom30 Amino Acid Analyzer, Biochrom, Cambridge, UK).

### 3.6. Cell Culture and Treatment with Blue Shark Collagen Hydrolysate

Adult human dermal fibroblasts (P10858 HDFa from Innoprot, Derio, Spain) were seeded into 96 well plates (passage three) at a density of 5 × 103 cells/well in 100 µL of Fibroblast Medium (FM) and incubated at 37 °C, 95% of humidity and 5% CO_2_, in a temperature-regulated incubator for 24 h. The medium was then removed and substituted by 100 µL of FM medium containing different concentrations (0.125, 0.25, 0.50, and 1 mg/mL) of commercial hydrolyzed collagen (CHC) and blue shark collagen hydrolysate (BSCH-C, see Table 2). Each hydrolysate concentration was seeded by sextuplicate and incubated for 24 h, 48 h, and 72 h. Likewise, six wells were left untreated as a control.

### 3.7. Cell Viability Assay

Cell viability was performed after the incubation periods of 24, 48, and 72 h, respectively, using the PrestoBlue™ Cell Viability Reagent (Invitrogen by Life Technologies, Carlsbad, CA, USA), a ready-to-use reagent for rapidly evaluating the viability and proliferation of cells based on cell metabolism following the manufacturer´s instructions. PrestoBlue^®^ reagent is reduced from resazurin, a blue compound, to resorufin, which is red in color. The conversion is proportional to the number of metabolically active cells and, therefore, can be measured quantitatively by absorbance. Plates were read in a Synergy MX Microplate Reader (Biotek, Winooski, VT, USA) at 600 nm (resazurin) and 570 nm (resorufin). Cell viability was normalized using the viability of the control wells for each incubation period.

### 3.8. RNA Isolation and Quantitative Real-Time Reverse Transcriptase-Polymerase Chain Reaction (RT-PCR)

Total RNA from each well was extracted with Cells-to-CT 1-Step TaqMan Kit (Thermofisher, Vilnius, Lithuania). Briefly, once the collagen hydrolysate medium was removed, cells were rinsed with 100 µL of cold phosphate buffer saline (PBS) two times. After this, 50 µL of the lysis buffer provided with the kit was added to each well and mixed thoroughly with the cells, leaving the mixture to stand for 5 min at room temperature. Then, 10 µL of the stop solution were added to each well, mixed, and incubated for 2 min. Finally, RNA measurement was performed by fluorimetry using Qubit v3.0 fluorimeter (Life Technologies, Eugene, OR, USA) with the Qubit RNA HS assay kit. RNA extracts were stored at −80 °C until the RNA expression assays were performed.

The real-time polymerase chain reaction (RT-PCR) was used to measure the expression of COL-I gene. Expression of the housekeeping gene, glyceraldehyde 3-phosphate dehydrogenase (GAPDH), was used as a reference. The primers and MGB-probes sequences are as follows: GAPDH-F: 5′-GGAAGCTCACTGGCATGGC-3′, GAPDH-R: TAGACGGCAGGTCAGGTCCA, GAPDH-P (probe): 5′-VIC-CCCCACTGCCAACGTGTCAGTG–MGB-3′, COL_I-F: 5′-ATGCCTGGTGAACGTGGT-3′, COL_I-R 5′-AGGAGAGCCATCAGCACCT-3′, COL_I-P: 5′ 6-FAM-ACCAGCATCACCTCTGTC-MGB-3′.

Real-time PCR assays were carried out in a 7500 Fast Real-Time PCR System equipment (Applied Biosystems, Waltham, MA, USA) using the TaqMan^®^ 1-Step qRT-PCR Mix. Reactions were performed in a total volume of 20 μL in a MicroAmp fast optical 96-well reaction plate. Each reaction contained 10 ng of RNA, TaqMan^®^ 1-Step qRT-PCR Mix (1×), water and a final concentration of 600 nM for each COL-I primers, 400 nM for each GAPDH primers and 200 nM for both probes. The following thermal cycling protocol was applied: 50 °C for 5 min; then 20 s at 95 °C, and 40 cycles at 95 °C for 15 s followed by 1 min at 60 °C. RT-PCR data were analyzed using the ΔΔCt method and normalized to the GAPDH values.

### 3.9. Human Pro-Collagen I Quantification from Fibroblast Cell Culture Supernatants by Sandwich ELISAs

Collagen hydrolysate medium was removed from polypropylene 96 well-plates and centrifuged at 2000× *g* for 10 min at 4 °C. Supernatants were diluted at 1:200, 1:800, and 1:1600 depending on the cell incubation time, 24, 48, and 72 h, respectively. Then, the amount of pro-collagen I was determined by ELISA employing the kit “Human Pro-Collagen I α 1/OLIA1” (R&D Systems, Abingdon, UK) following the manufacturer’s instructions, including some modifications in the incubation conditions with the capture and detection antibody (1 h at 37 °C instead of 2 h at room temperature). Finally, plates were read using a spectrophotometer (Synergy Mx from Biotek Instruments, Winooski, VT, USA) at 450 nm with a wavelength correction set to 540 nm. For data analysis, the software “Four Parameter Logistic Curve” online data analysis tool was employed [25].

### 3.10. Cell Viability, RNA Expression, and Pro-Collagen I Biosynthesis Statistical Analysis

All values were expressed as a mean value and standard deviation (SD) of six independent samples (*n* = 6). Statistical analysis was performed with IBM SPSS 26 software (IBM Corporation, Armonk, NY, USA) using one-way ANOVA followed by HSD Tukey test or by Welch ANOVA followed by Games-Howell when non-uniform variances were appreciated. For data distributed with non-normality, the non-parametric test Kruskal– Wallis was employed. *p*-values < 0.05 were considered statistically significant. Correlations between every two variables were assessed using Pearson’s correlation coefficient (**: correlation is significant at *p* < 0.01 level).

## 4. Conclusions

GPC-LS methodology resulted in being suitable for analyzing the Mw of blue shark collagen hydrolysates prepared in different hydrolysis conditions and permitted to select those conditions of hydrolysis leading to obtaining the highest molecular weight hydrolysate. The highest blue shark molecular weight hydrolysate and a commercial hydrolysate were analyzed in terms of cell viability and collagen expression at the gene level by determining the mRNA expression of the Pro-collagen I gene (COL_I) in a fibroblast cell culture. Results revealed a stimulatory effect of BSCH on fibroblast metabolism, leading to both an increase in mRNA and pro-collagen I biosynthesis without observing an increase in cell proliferation. This stimulatory effect was 2.3 times higher than that of untreated fibroblasts and higher than that achieved with commercial hydrolysate. The difference in overexpression might probably be explained in relation to the amino acid and molecular weight different profiles shown by these two hydrolysates. Finally, BSCH-C did not show a cytotoxicity effect on fibroblasts, so it could be a potential candidate to stimulate biosynthesis processes in the dermal cells that produce protein components present in the extracellular matrix.

## Figures and Tables

**Table 1:** The proximate composition of lyophilized BSASC extracted from the skin.

**Lyophilized BSASC**	**Proximate Composition (%)**		
	**Moisture**	**Protein**	**Ash**
	12.93 ± 0.70	84.46 ± 0.65	1.32 ± 0.54

**Table 2:** Mw (Mean ± SD) obtained after GPC-LS analysis of BSCH prepared in different conditions (enzyme: Alcalase or Papain; time of hydrolysis: 30 min or 6 h; E/S ratio: 1/5 or 1/20). Values are resultant of three GPC-LS determinations per hydrolysis condition.

Blue Shark Collagen Hydrolysis Conditions				Mw (kDa)
Experiments	Enzyme	E/S Rate	Time (h)	
**A**	Papain	1/5	6	11.6 ± 5.4
**B**	Alcalase	1/20	6	3.5 ± 0.2
**C**	Papain	1/20	0.5	34.43 ± 10.2
**D**	Alcalase	1/20	0.5	3.2 ± 0.7
**E**	Alcalase	1/5	6	8 ± 5.0
**F**	Papain	1/5	0.5	20.72 ± 4.2
**G**	Papain	1/20	6	9.26 ± 5.3
**H**	Alcalase	1/5	0.5	4.3 ± 0.0

**Table 3:** The molecular weight distribution of BSCH and CHC using the SEC technique. Values are expressed as percentages of the areas in the chromatograms. Letters A–H indicate different conditions of hydrolysis, which could be consulted in Table 2.

Retention Time (min)	Mw (kDa)	% Area								
		BSCH								CHC
		A	B	C	D	E	F	G	H	
20–30	17–3	37 ± 7	2 ± 0	9.33 ± 2	4 ± 1	3.5 ± 2	18.3 ± 7	30 ± 0	2.67 ± 2	50.6 ± 0.6
30–40	3–0.5	52 ± 9	84.5 ± 3	71.7 ± 3	88.6 ± 1	82 ± 2	69.3± 6	55 ± 13	89 ± 2	42.4 ± 0.2
40–50	0.5–0.09	11 ± 2	13 ± 4	19 ± 2	7.3 ± 2	14.5 ± 4	12.3 ± 4	15 ± 14	8.33 ± 2	6.9 ± 0.4

**Table 4:** Amino acid composition of BSCH-C and CHC. Values are expressed in residues per 1000 residues.

Amino Acid	BSCH-C	CHC
Hydroxyproline	70.249 ± 0.05	89.85 ± 0.33
Aspartic acid	44.761 ± 0.02	43.27 ± 0.07
Threonine	23.567 ± 0.02	15.91 ± 0.06
Serine	43.928 ± 0.07	35.31 ± 0.12
Glutamic acid	76.674 ± 0.08	70.18 ± 0.05
Proline	112.081 ± 0.08	115.41 ± 0.67
Glycine	306.533 ± 0.32	296.87 ± 1.48
Alanine	118.869 ± 0.04	106.385 ± 0.31
Cysteine	3.696 ± 0.004	3.383 ± 0.11
Valine	24.817 ± 0.030	20.650 ± 0.20
Methionine	14.3 ± 0.006	6.137 ± 0.00
Isoleucine	21.357 ± 0.04	11.158 ± 0.00
Leucine	24.856 ± 0.02	23.058 ± 0.03
Tyrosine	4.4 ± 0.01	2.086 ± 0.02
Phenylalanine	16.699 ± 0.06	12.299 ± 0.05
Hydroxylysine	4.518 ± 0.02	5.977 ± 0.04
Histdine	7.543 ± 0.002	5.543 ± 0.016
Lysine	28.206 ± 0.06	27.158 ± 0.08
Arginine	52.948 ± 0.02	46.090 ± 0.16
